# PD-ResNet for Classification of Parkinson’s Disease From Gait

**DOI:** 10.1109/JTEHM.2022.3180933

**Published:** 2022-06-08

**Authors:** Xiaoli Yang, Qinyong Ye, Guofa Cai, Yingqing Wang, Guoen Cai

**Affiliations:** School of Information EngineeringGuangdong University of Technology 47870 Guangzhou 510000 China; Department of NeurologyFujian Medical University Union Hospital 117890 Fuzhou 350001 China

**Keywords:** Gait motor disorder, improved focal loss function, PD-ResNet, Parkinson’s disease (PD)

## Abstract

Objective: To develop an objective and efficient method to automatically identify Parkinson’s disease (PD) and healthy control (HC). Methods: We design a novel model based on residual network (ResNet) architecture, named PD-ResNet, to learn the gait differences between PD and HC and between PD with different severity levels. Specifically, a polynomial elevated dimensions technique is applied to increase the dimensions of the input gait features; then, the processed data is transformed into a 3-dimensional picture as the input of PD-ResNet. The synthetic minority over-sampling technique (SMOTE), data augmentation, and early stopping technologies are adopted to improve the generalization ability. To further enhance the classification performance, a new loss function, named improved focal loss function, is developed to focus on the train of PD-ResNet on the hard samples and to discard the abnormal samples. Results: The experiments on the clinical gait dataset show that our proposed model achieves excellent performance with an accuracy of 95.51%, a precision of 94.44%, a recall of 96.59%, a specificity of 94.44%, and an F1-score of 95.50%. Moreover, the accuracy, precision, recall, specificity, and F1-score for the classification of early PD and HC are 92.03%, 94.20%, 90.28%, 93.94%, and 92.20%, respectively. Furthermore, the accuracy, precision, recall, specificity, and F1-score for the classification of PD with different severity levels are 92.03%, 94.29%, 90.41%, 93.85%, and 92.31%, respectively. Conclusion: Our proposed method shows better performance than the traditional machine learning and deep learning methods. Clinical impact: The experimental results show that the proposed method is clinically meaningful for the objective assessment of gait motor impairment for PD patients.

## I. Introduction

Parkinson’s disease (PD) is a progressive, neurodegenerative disease resulting from the degenerative death of dopaminergic neurons. PD patients often suffer from motor impairment, e.g., resting tremors, muscle rigidity, bradykinesia, and postural imbalance, since dopaminergic neuromodulation can precisely impact motor control [1]. In addition, PD patients also experience non-motor symptoms such as depression, constipation, and sleep disturbances. Both motor and non-motor symptoms can negatively affect patients’ life in numerous aspects. Timely detection and early treatment can effectively prevent motor complications and improve the quality of life for PD patients [2]. Therefore, it requires the diagnosis as early as possible to prevent and control PD.

Traditionally, the clinical assessment scales are used to assess PD patients, which faces two apparent flaws. On the one hand, parkinsonian features may be so subtle at the beginning of the disease that leads to the misdiagnosis [3]. On the other hand, due to the complexity of the evaluation, a professional and experienced neurologist is required to conduct a comprehensive evaluation, which consumes more time and cost. Fortunately, the typical motor symptoms of PD patients can be objectively quantified by using some wearable sensor devices [4]. In this paper, the GYENNO MATRIX wearable device is used to extract the gait features of subjects for the identification of PD [5]. GYENNO MATRIX is a motor evaluation device that includes ten wireless MEMS inertial sensor nodes, a data center, and corresponding computer software. The data of the sensors can be transmitted to the computer software in real time through the data center, and the computer software can automatically quantify the human gait parameters.

Wearable sensor devices enable PD patients to be measured anytime and anywhere, and they can subsequently generate many gait indicators associated with the quantitative assessment of mobility [6]. Eight spatial-temporal and kinematic gait features in 49 subjects were extracted using two shank-mounted inertial measurement units in [7]. Then, an support vector machine (SVM) based classifier for the gait analysis and classification was developed to achieve an overall accuracy of 93.9%. Referring to the study by Sama *et al.*, bradykinetic was evaluated by analyzing the signals from a triaxial accelerometer placed on the waist of 12 PD patients [8]. Most of the studies suffered from a small number of sensors and small sample sizes. Referring to the study by Mirelman *et al.*, 134 gait features from 332 PD patients and 100 HCs were extracted via multiple wearable sensor devices [9]. Then the traditional machine learning (ML) methods were employed to classify the severity of PD. In this paper, we recruit 457 subjects and extract 194 comprehensive features from each subject under three measurement conditions. Subsequently, a polynomial elevated dimensions method is applied to increase the number of gait features. Synthetic minority over-sampling technique (SMOTE) [10]and data enhancement technology are employed to further expand the samples.

For one-dimensional (1-D) clinical datasets, there have extensive literatures, such as [7]–[9], to do manual feature extraction on the raw data before using traditional ML methods. However, it requires expert domain knowledge and it may influence the model’s accuracy. With the rapid development of artificial intelligence technologies, an increasing number of experts are devoting extensive research to the application of deep learning (DL) in the medical field [11], [12]. Compared with human experts, DL performs well in prediction and diagnosis. Convolutional neural network (CNN) is the main area of interest among DL, which has been widely used in image processing thanks to its powerful functions [13]. Several advanced CNN models were proposed, such as AlexNet [14], VGGNet [15], Google Inception Net (GoogLeNet) [16], and Residual Network (ResNet) [17]. Transferring the parameters from a pre-trained model to a new model is called transfer learning [18]. Many studies fine-tuned the ImageNet-based pre-trained model and subsequently applied the model to the field of medical imaging [19]–[21]. However, ImageNet is an image dataset about nature and is different from the medical image datasets. Blind and violent transfer on unrelated areas is likely to lead to the failure or even a negative transfer. Therefore, in this paper, we build and train neural network from scratch. What we do is not as simple as using pre-trained models in DL but requires more expertise and experience.

Since gait features in PD patients are not completely independent from each other, the 2D CNN is used in the paper to extract the spatial information among features. To our best knowledge, there is no literature on using ResNet to identify PD and HC from multiple gait features. The ResNet does well in classification tasks because it allows the neural network to go deeper, be more accurate, have fewer parameters, and solve the gradient problems. These advantages of the ResNet mainly benefit from the residual unit [17]. In this paper, considering the residual unit as the basic unit, a new model based on ResNet architecture, named PD-ResNet, is proposed to learn the gait differences between PD and HC. The main contributions of this paper are summarized as follows.

1) To improve the performance, the polynomial elevated dimensions technique is used to enhance the dimensionality of the features of the input data, and both SMOTE and data enhancement technology are adopted to increase the sample size.
2) A PD-ResNet model is built to learn the gait information of subjects to objectively classify PD and HC, early PD and HC, as well as early PD and moderate to advanced PD.
3) An improved focal loss function is proposed to focus on the learning of the samples that are difficult to be judged and to stop the learning in abnormal samples.

The rest of this paper is organized as follows. Section II introduces the dataset used and the details of the proposed method. The evaluation criteria, experimental details, and the final results of the experiments are described in Section III. We make a brief review and discussion of our work in Section IV. Section V provides the conclusion.

## II. Materials and Methods

### A. Database

This study has got the support of the Medical Ethics Committee of Fujian Medical University Union Hospital (Fuzhou, China). We recruited a total of 457 subjects. The dataset for this study was obtained from the hospital from November 2018 to August 2021. Based on the Movement Disorders Society (MDS) clinical diagnostic criteria for PD [22], movement disorder specialists included those subjects diagnosed with stages 1–3 of the Hoehn and Yahr’s scale (H-Y) [23] into PD patients. Of all subjects, 296 PD patients and 161 age-matched HCs were identified as the PD group and the HC group, respectively. Patients with any other neurological, psychiatric or orthopaedic diseases were excluded. The age-matched HCs were collected according to similar exclusion criteria. We further referred to the PD group with H-Y scores that are greater than 2.5 as the moderate to advanced PD group and the rest as the early PD group. H-Y stages were determined while patients were ON medication state, which occurred approximatively one hour after medication intake. The demographic and clinical information is described in Table 1. Before the gait features were measured, each subject was asked to complete three Unified Parkinsons Disease Rating Scale (UPDRS), namely UPDRS-I, UPDRS-II and UPDRS-III. To increase the number of the features and to extract comprehensive information, we extracted 190 gait features from each subject under three conditions and 4 demographic features (See Supplementary Tabel S1). The three testing conditions are as follows: 1) Time Up and Go (TUG): Stand up slowly from the chair, walk along the designated path for a certain distance as usual and then turn around and walk back to the original position. We extracted 97 gait features during this process. 2) TURN: We extracted seven gait features under the given way of spinning. 3) NARROW: Walk as usual in a narrow path. We extracted 86 gait features in this process. In addition, 4 demographic characteristics, i.e., gender, age, length of thighs, and length of lower legs, were also included in the list of features because these may independently influence gait [9]. Before testing, clinical staff or family members were arranged to instruct and assist the subjects in putting on the wearable sensors and completing the standardized movements. Furthermore, to ensure the validity of the results, each task was required to perform 2–3 times, then the test results were averaged.

**TABLE 1:** Demographic and Clinical Information of the Subjects

Characteristic	Early PD (H-Y1–2.5) ( $n=230$) $\mu \pm \sigma $	Moderate to advanced PD (> H-Y2.5) ( $n=66$) $\mu \pm \sigma $	HC ( $n=161$) $\mu \pm \sigma $	$p$ value between early PD and moderate to advanced PD	$p$ value between early PD and HC	$p$ value between PD and HC
Age[years]	64.46 ± 10.27	66.94 ± 9.31	62.55 ± 8.71	0.079	0.048	0.007
Sex[male / female] ( $n$)	143 / 87	29 / 37	66 / 95	0.008	$p < 0.001 $	$p < 0.001 $
Height [cm]	163.53 ± 7.87	160.68 ± 8.65	162.03 ± 6.74	0.020	0.081	0.3
UPDRS UPDRS-I	2.45 ± 1.77	3.17 ± 1.89	–	0.005	–	–
UPDRS-II	9.32 ± 4.29	14.58 ± 6.70	–	$p < 0.001$	–	–
UPDRS-III	23.43 ± 12.78	32.85 ± 13.59	–	$p < 0.001$	–	–
Disease duration [months]	54.31 ± 44.01	67.79 ± 41.05	–	0.032	–	–
LEDD [mg / day]	407.90 ± 240.43	478.17 ± 266.91	–	0.042	–	–

$\mu$ and $\sigma$ represent the mean and standard deviation, respectively. T-test was applied to check the differences in age, height, UPDRS, disease duration and LEDD. The gender differences were compared via chi-square analysis, $p < 0.05$ indicates a statistically significant difference. UPDRS stands for the Unified Parkinson’s Disease Rating Scale. LEDD is the levodopa equivalent daily dose.

### B. Method Outline

**FIGURE 1.**
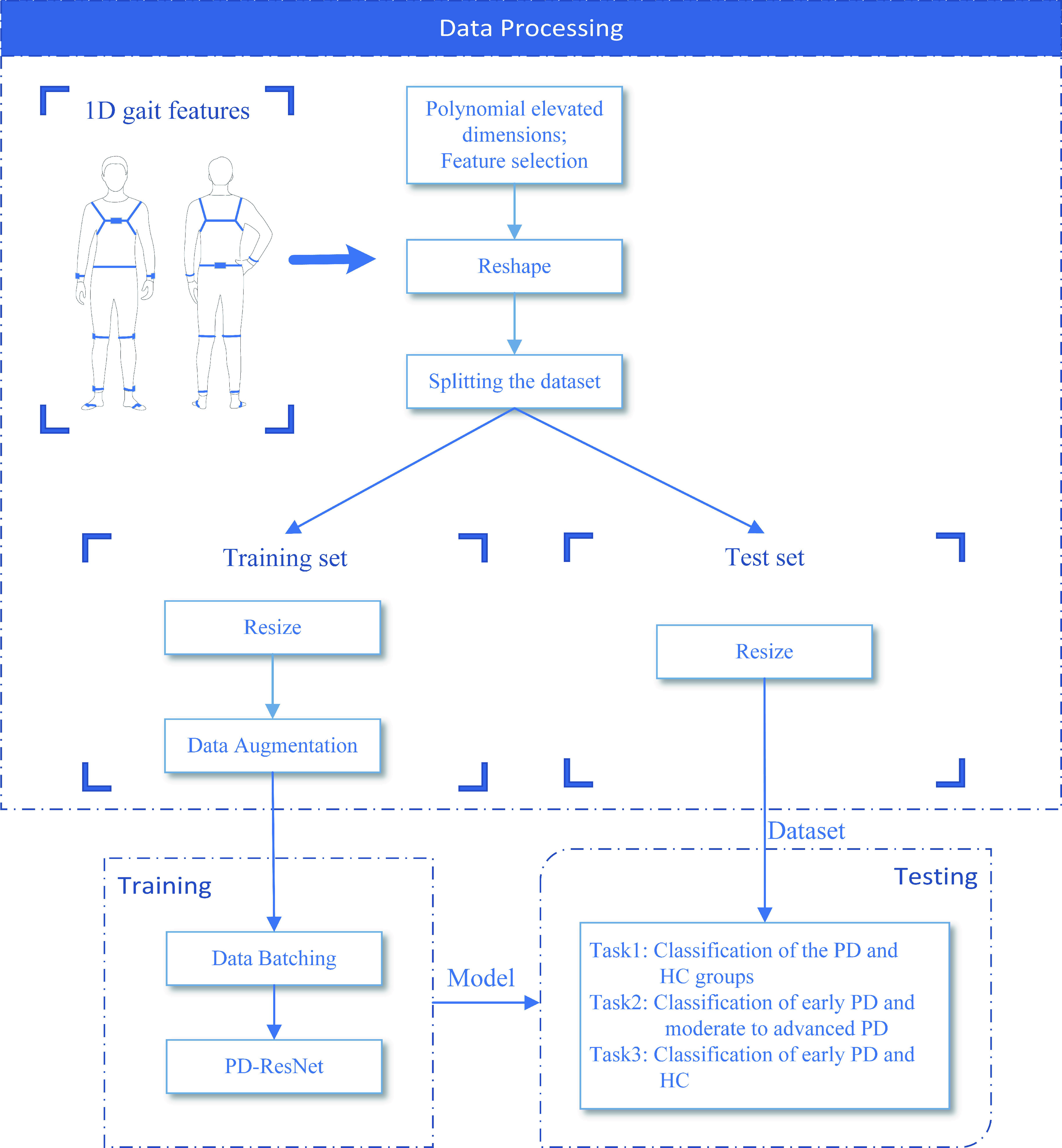
The flowchart of our proposed approach.

In this paper, a two-dimensional (2-D) CNN model is applied. 2-D CNN model is commonly used in computer vision and image processing, where the input layer receives only 2-D or three-dimensional (3-D) arrays. To this end, the gait data should be first converted from one-dimensional (1-D) arrays to 3-D arrays. Fig. 1 illustrates the flowchart of the proposed approach. Transformation of data dimensions was completed during data pre-processing. The sample imbalance between PD group and HC group was solved by SMOTE, the feature dimensionality of the input dataset was raised by a polynomial elevated dimensions technique, and the dataset was subsequently transformed into the 3-D images. We randomly divided the dataset into two groups, i.e., a training set and a test set. The training set, after data augmentation, was fed into the PD-ResNet for batch training. In addition, the Root Mean Square Propagation (RMsprop) optimization algorithm, learning rate (LR) decay technique, early stopping technique, and improved focal loss function were employed to achieve excellent training results and generalization ability. Finally, the model performance was validated by a test set.

### C. Data Pre-Processing

#### 1) Sample Balancing

The initial samples consisted of 296 PD patients and 161 HCs. Since sample imbalance could negatively affect the classification performance and model’s accuracy [24], it needs to deal with the initial imbalanced samples. The dominant approach of overcoming the class imbalance in almost all analytic scenarios is oversampling [25]. Random minority oversampling methods replicate samples from minority classes, which may cause overfitting [10], [24]. As a modified scheme of the random oversampling algorithm, SMOTE is a technique for manually synthesizing new samples based on a minority class, which is suitable for tasks with input datasets in the type of tabular or vector [26]. The SMOTE increases the samples by using random interpolation between minority class samples according to the sample imbalance ratio. To realize sample balance and increase the amount of data, SMOTE generates some additional HC samples.

#### 2) Polynomial Elevated Dimensions

To transform 1-D data into 3-D data and get a large size, it needs to enhance the feature dimensionality of the input data. The polynomial elevated dimensions technique, which uses the interactive multiplication of feature data to increase its dimensionality, has become a simple and effective method. In this method, the features are mapped to a high dimensional space, thus obtaining the data projection in the space. In this paper, the polynomial elevated dimensions technique is utilized to adjust the dataset, then the processed dataset is transformed into the type of $1 \times 117 \times 117$.

#### 3) Data Augmentation and Batch Processing

The dataset is first normalized to improve the convergence speed and model’s accuracy. The dimensions of the processed dataset are resized into $1 \times 112 \times 112$. 70% of the dataset is adopted as a training set, and 30% is used as a test set to evaluate the model’s effectiveness. Because deep neural networks need to rely on a sufficient number of data samples for training to avoid overfitting, a relatively low number of data is a common challenge. Data augmentation can increase the diversity of the dataset and reduce the possibility of overfitting during training. In this paper, in addition to using the SMOTE to expand the dataset, the random erasing data augmentation technique [27] is also utilized to further increase the number of the data samples on the training set. We batch the data samples, each containing 23 samples^1^, to reduce computational complexity and prevent the loss function from jumping into local minima during training. Furthermore, to realize online data enhancement, random erasing starts only before each small batch of the dataset enters the model.^1^Batch size is actually a hyper parameter that can be adjusted according to the specific situation. In this paper, considering the balance between generalization ability and the performance of the model, the optimal batch size is chosen to be 23.

### D. Residual Network

**FIGURE 2.**
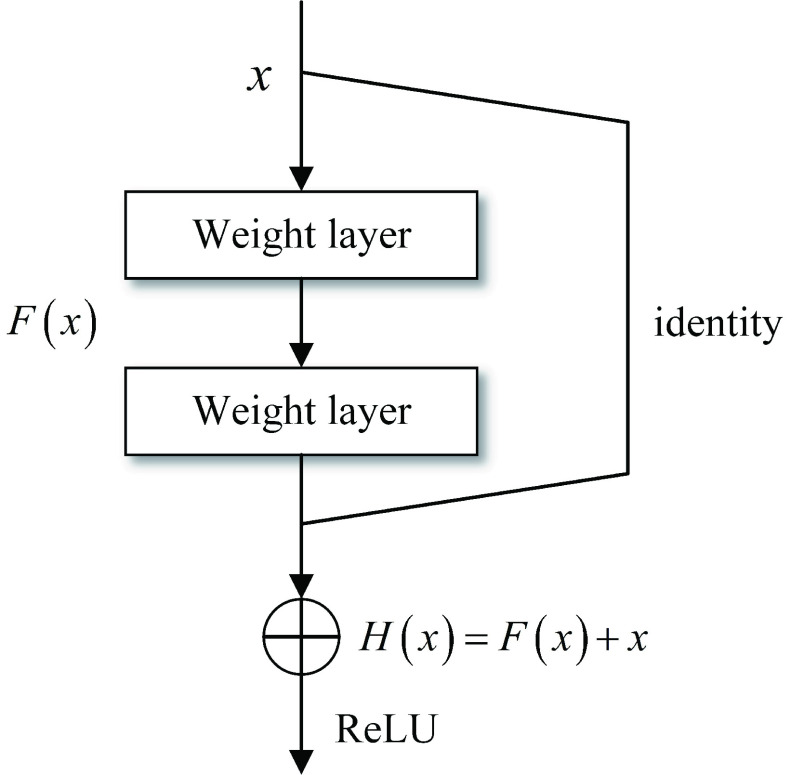
The basic structure of the resnet unit.

ResNet can provide excellent performance in various applications, such as image classification, image generation, visual recognition, natural language processing, speech recognition and user prediction. Fig. 2 illustrates the basic structure of the residual unit [17]. $H(x)$ and $F(x)$ represent the underlying mapping of the input value $x$ after two branches and the residual mapping of $x$ after two weight layers, respectively. It can be seen that the residual unit turns the issue from fitting the relationship between $H(x)$ and $x$ to the relationship between $F(x)$ and $x$ by adding an identity function as the shortcut connection. Concerning the residual network, two distinct advantages are presented compared to the common CNN as follows.

#### 1) Easier to be Optimized

Generally, the activation function before the output layer of the residual unit is ReLU activation function, which acts as a constant function of 0 or an identity function. We stacked multiple residual units together for learning. Assuming that the ReLU activation functions are identity functions, one has

\begin{equation*} {x_{l + 1}} = {x_{l}} + F\left ({{x_{l},\left \{{ {W_{l}} }\right \}} }\right),\tag{1}\end{equation*}

where $x_{l}$ represents the input of the $l$th resnet unit, and $W_{l}$ denotes the weights. $x_{l+1}$ and $F(x_{l},\left \{{ {W_{l}} }\right \})$ represent a direct forward propagated output and the residual mapping to be learned of the $l$th resnet unit, respectively.

Then the forward propagation process of the residual network can be defined as

\begin{equation*} {x_{L}} = {x_{l}} + \sum \limits _{i = l}^{L - 1} {F\left ({{x_{i},\left \{{ {W_{i}} }\right \}} }\right)},\tag{2}\end{equation*}

where $x_{L}$ denotes the accumulated output of $L-1$ connected residual units.

However, for a common CNN, its forward propagation process can be described as

\begin{equation*} {x_{L}} = {x_{l}}\prod \limits _{i = l}^{L - 1} {W_{i}},\tag{3}\end{equation*}

where $W_{i}$ specifies the weights, $x_{l}$ and $x_{L}$ represent the input of the $l$th convolutional layer and the output after $L-1$ convolutional layers, respectively.

By comparing Eq. (2) with Eq. (3), it can be yielded that the residual network is less computationally intensive and easier to be optimized than the common CNN.

#### 2) A Better Solution to the Gradient Problem

Referring to Eq. (2), the gradient of the residual network can be expressed in the backpropagation process as

\begin{equation*} \frac {\partial E}{{\partial {x_{l}}}} = \frac {\partial E}{{\partial {x_{L}}}}\left ({{1 + \frac {\partial }{{\partial {x_{l}}}}\sum \limits _{i = l}^{L - 1} {F\left ({{x_{i},\left \{{ {W_{i}} }\right \}} }\right)} } }\right),\tag{4}\end{equation*}

where $E$ denotes the loss function of the model. The gradient of the common CNN can be given by

\begin{equation*} \frac {\partial E}{{\partial {x_{l}}}} = \frac {\partial E}{{\partial {x_{L}}}}\prod \limits _{i = l}^{L - 1} {W_{i}}.\tag{5}\end{equation*}

By comparing Eq. (4) and Eq. (5), we can conclude that when the network deepens, the common CNN is prone to the problems of gradient disappearance and gradient explosion, while the ResNet can well solve such issues.

### E. Proposed PD-ResNet

**FIGURE 3.**
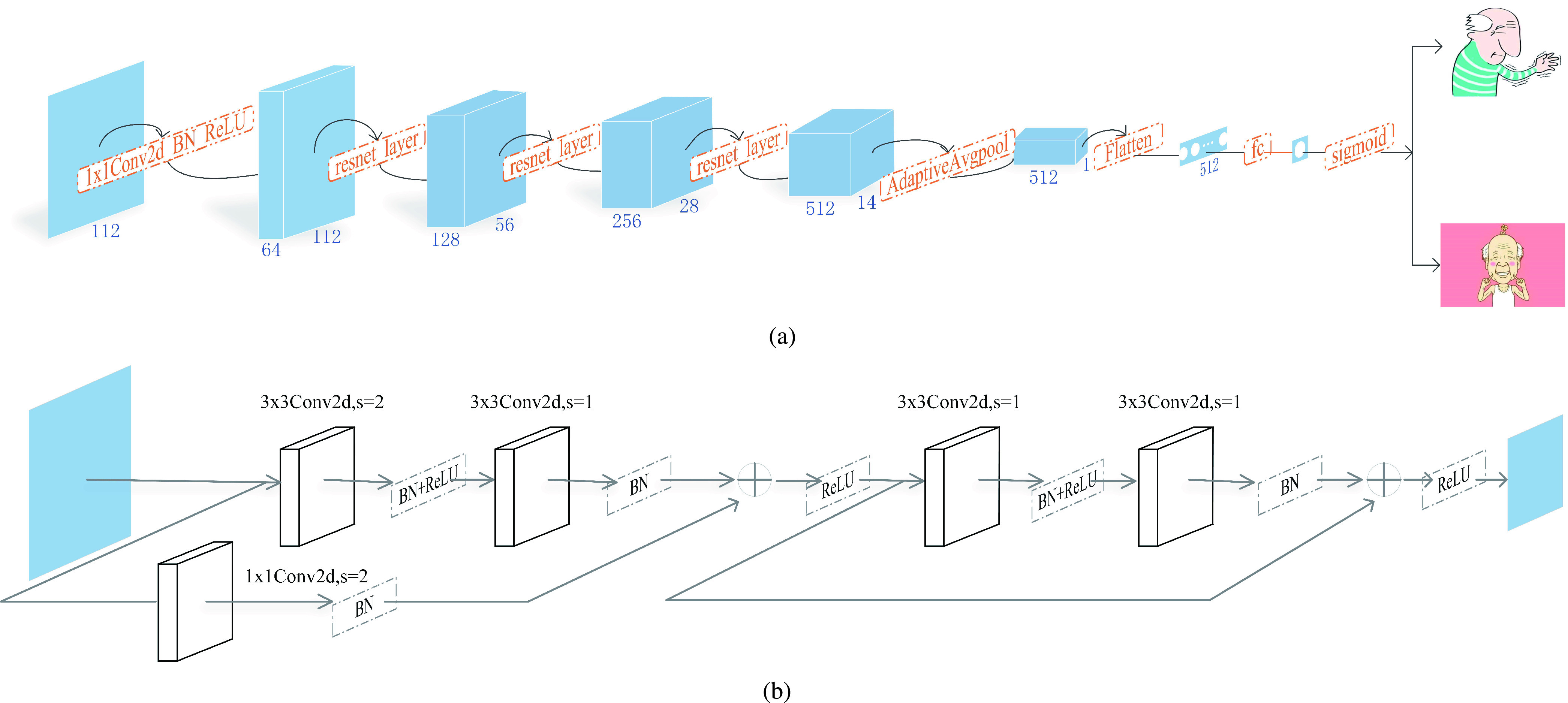
The architecture of our proposed model. (a) The structure of PD-ResNet; (b) The structure of the resnet layer.

The proposed PD-ResNet is shown in Fig. 3 (a), which is constructed from scratch using residual units as the basic units. The dataset with 1 $\times112\,\,\times112$ pixels is fed into the network, and a fine feature mapping can be gained after a 1 $\times $ 1 convolution is carried out. If the number of the hidden layers is too much, gradient problems may occur during training; on the contrary, the number that is too less stops the model from learning in the optimal direction. Usually, the number of hidden layers is regarded as a hyperparameter from the perspective of the model. By several experiments, we found three resnet layers for three downsamplings, which is a reasonable choice between performance gain and computational complexity. The resnet layer is shown in Fig. 3 (b). We exploit AdaptiveAvgPool2d to convert the size of the image to 512 $\times1\,\,\times1$. AdaptiveAvgPool2d extracts deeper feature information and reduces the number of parameters and the computational complexity of the network. Then, the linear layer is employed as a classifier, and the number of the output neurons corresponds to the number of classes, i.e., 2. Afterward, sigmoid activation function is carried out to normalize the classification results. In the classification of HC versus (vs.) PD, early PD vs. moderate to advanced PD, and HC vs. early PD, a classification result higher than or equal to 0.5 is considered as PD, moderate to advanced PD and early PD, respectively.

### F. Improved Focal Loss

The cross-entropy loss function effectively measures weak variation, and it converges quickly, thus making it one of the most popular loss functions within classification networks. Mathematically, it can be given by

\begin{align*} CE\left ({{ p _ {+},y} }\right)=\begin{cases} \displaystyle {- \log \left ({{ p _ {+} } }\right)},& y=1\\ \displaystyle { - \log \left ({{1 - {{p _ {+} }}} }\right)},&y=0,\\ \displaystyle \end{cases}\tag{6}\end{align*}

where $CE$ is the loss value, $p_{+}$ represents the probability of a positive prediction, and $y$ denotes the real value of the sample.

We define the following equation as

**FIGURE 4.**
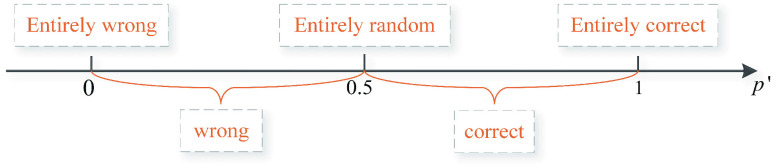
The relationship between $p'$ and the predicted results.

\begin{align*} p'=\begin{cases} \displaystyle { p _ {+} },& y=1\\ \displaystyle {1-{ p _ {+} }},& y=0,\\ \displaystyle \end{cases}\tag{7}\end{align*}

where $p'$ represents the degree of correct prediction. $p' = 0$ means that the prediction result is entirely wrong, and $p' = 1$ when the prediction result is entirely correct. The predicted result is considered to be completely random when $p' = 0.5$. The relationship between $p'$ and the predicted results is depicted in Fig. 4. The numerical axis being closer to the left indicates that the sample is more difficult to be judged (which is defined as a hard-to-judge sample), while the numerical axis be closer to the right denotes that the sample is easier to be judged (which is defined as an easy-to-judge sample).

Combining Eq. (6) with Eq. (7), the cross-entropy loss function is written by

\begin{equation*} CE\left ({{p',{\text {y}}} }\right) = - \log p'.\tag{8}\end{equation*}

To get more hard-to-judge samples to be trained, the weight of the loss value should be increased, and conversely, the weight of that should be reduced for the easy-to-judge samples. The basic idea of focal loss function is that a dynamic coefficient is added in the loss function to achieve the adjustment [28]. The expression for focal loss is given by

\begin{equation*} FL\left ({{p'} }\right) = - {\left ({{1 - p'} }\right)^\gamma }\log \left ({{p'} }\right),\tag{9}\end{equation*}

where $FL$ implies the loss value and $\gamma $ is a hyperparameter.

It is apparent that the dynamic coefficient ${\left ({{1 - p'} }\right)^{2}}$ is a concave function. This paper needs to strengthen the training of samples with incorrect judgments while weakening the learning of samples with correct judgments. As a result, let the loss value fall more slowly when $p'$ is in the range from 0 to 0.5, and let it fall more quickly when $p'$ is in the range from 0.5 to 1. The dynamic coefficient is set up as a convex function, which do not need to be calculated in a strict sense. Then the loss function is written as

\begin{equation*} FL\left ({{p'} }\right) = \left ({{ - p{'}^{2} + 1} }\right) \times \left [{ { - \log \left ({{p'} }\right)} }\right].\tag{10}\end{equation*}

However, some anomalous samples exist due to misjudgments, mishandling during checking, improper data augmentation, etc. The most extreme case is that $p'=0$. Learning from these samples may be counterproductive. This paper sets a threshold, and the sample is treated as an outlier when $p'$ is less than the threshold. The loss value under the anomalous sample is set to zero. The threshold is taken to be 0.05 in the experiment. An improved focal loss method is proposed, which is given by

\begin{align*} {\text {IFL}}\left ({{p'} }\right)=\begin{cases} \displaystyle {0}, &p' < 0.05\\ \displaystyle {\left ({{ - p{'}^{2} + 1} }\right) \times \left [{ { - \log \left ({{p'} }\right)} }\right]},& otherwise. \\ \displaystyle \end{cases}\!\!\! \\\tag{11}\end{align*}

The loss value for each epoch is the average of the IFL for each sample in that epoch.

## III. Experiments and Results

### A. Evaluation Metrics

**FIGURE 5.**
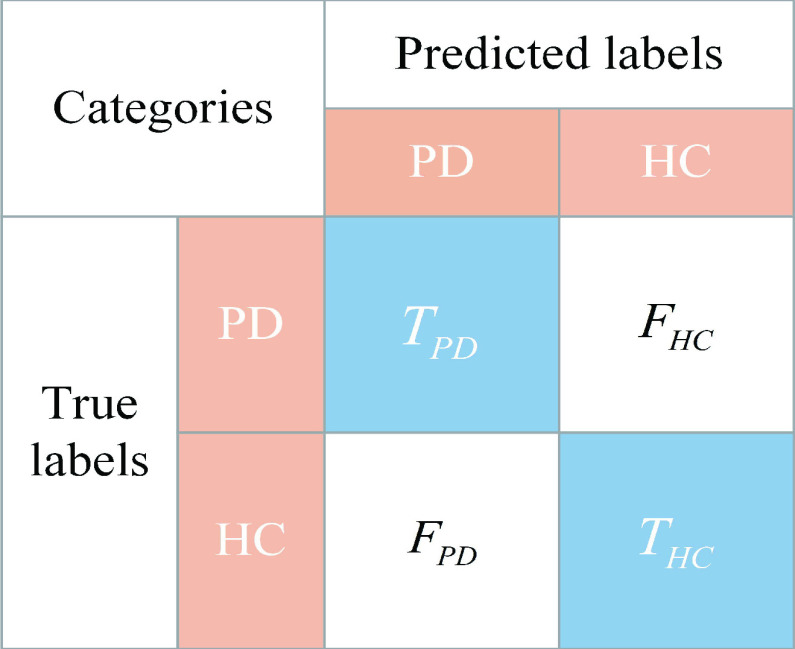
The structure of the confusion matrix.

In this paper, a confusion matrix is used to evaluate the performance of the proposed model, which can measure the accuracy of a classifier. Fig. 5 exhibits the structure of the confusion matrix, which shows the details of the classification results on PD vs. HC. True HC ( $T_{HC}$) and true PD ( $T_{PD}$) represent the number of HC and PD samples that are correctly classified, respectively. The false HC ( $F_{HC}$) indicates the number of PD samples that are incorrectly predicted as HC, and the false PD ( $F_{PD}$) indicates the number of HC samples that are incorrectly predicted as PD. Hence, the values on the diagonal line, i.e., in the blue boxes, indicate the number of correctly classified samples, while the other values are the number of incorrectly classified samples. Accuracy (Acc), precision (Pre), recall (Rec), specificity (Spe) and F1-score (F1) can be calculated separately by a confusion matrix, which serve as evaluation metrics for the proposed model. These indicators are defined as

\begin{align*} {\text {Acc}}=&\frac {{{T_{HC}} + {T_{PD}}}}{{{T_{HC}} + {T_{PD}} + {F_{PD}} + {F_{HC}}}} \times 100\%, \tag{12}\\ \text {Pre}=&\frac {{{T_{PD}}}}{{{T_{PD}} + {F_{PD}}}} \times 100\%, \tag{13}\\ \text {Rec}=&\frac {{{T_{PD}}}}{{{T_{PD}} + {F_{HC}}}} \times 100\%, \tag{14}\\ \text {Spe}=&\frac {{{T_{HC}}}}{{{T_{HC}} + {F_{PD}}}} \times 100\%, \tag{15}\\ \text {F1}=&\frac {{2 \times \text {Pre}\times \text {Rec}}}{{\text {Pre} + \text {Rec}}}.\tag{16}\end{align*}

### B. Experimental Details

Both PD-ResNet and GoogLeNet are implemented under the DL framework of PyTorch. SVM, eXtreme Gradient Boosting (Xgboost), and Random Forest (RF) are implemented using the scikit-learn ML library. All programs are written and implemented using python 3.7. During the training process, the value of the loss function is minimized as much as possible to achieve optimal results. The initial learning rate is set as 10^−3^, and it decays to one-fifth of the previous value for every ten epochs run. The learning rate decay method aims to speed up the decline of loss value at the beginning of training and prevent the learning rate from being so large that the loss value falls into a local minimum in the later stages of training. The batch size is 23. We chose the RMSprop optimization algorithm as the optimization strategy that provides a better result during exploratory tests. Besides, we use the early stopping technique to prevent the model from overfitting. Finally, the training process ends on the 25th epoch.

The same balanced dataset and evaluation metrics are used for all comparative classifiers. The evaluation of ML experimental results is carried out via ten fold cross-validation. In each fold, 90% of the data samples are used for model training, and 10% are used for model validation. To save computational resources, for DL experiments, 70% of the dataset is adopted as a training set, and 30% is used as a test set. All the comparative classifiers are optimized, and the optimal results are selected. A random number seed is set to allow for full reproducibility of the experimental results.

### C. Classification Results

#### 1) Classification of PD and HC

**FIGURE 6.**
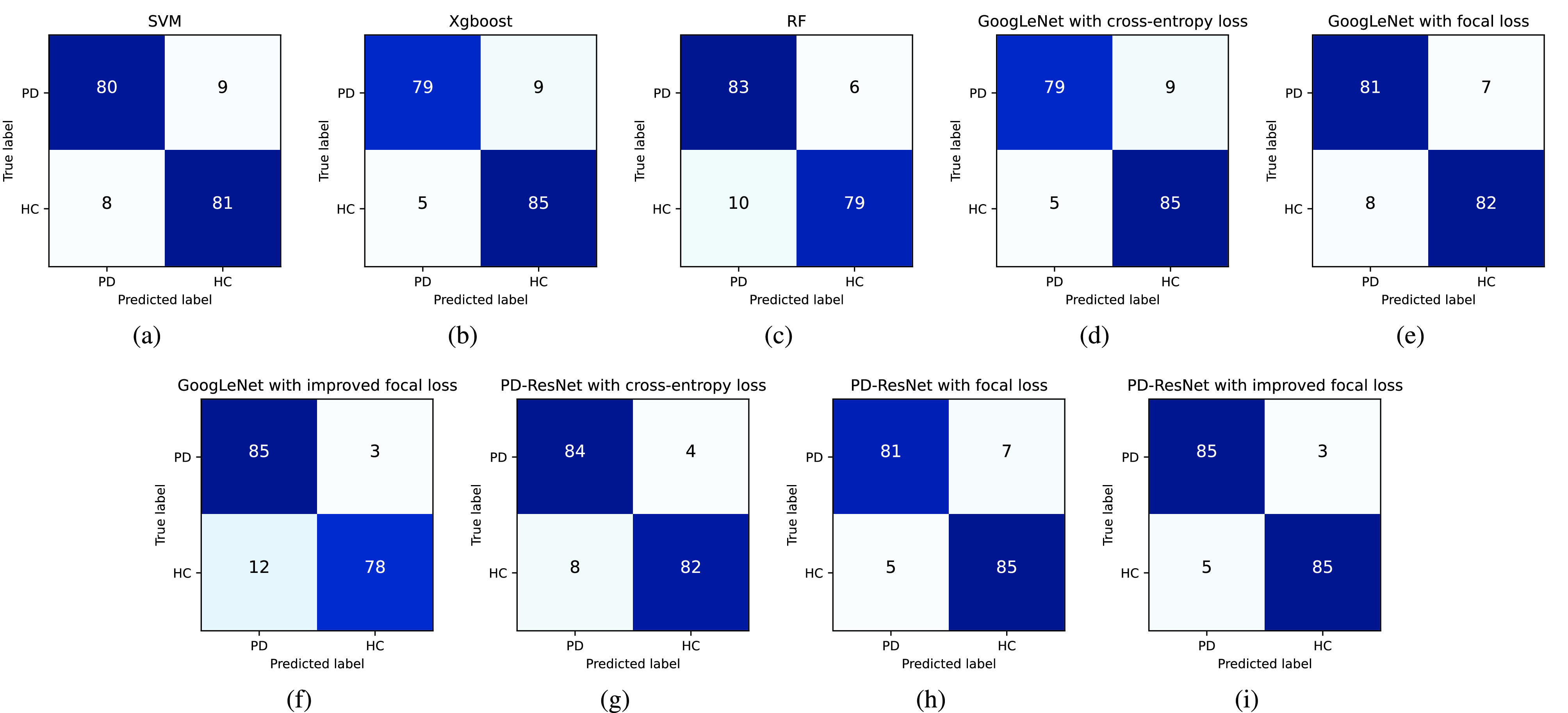
Confusion matrices for all mentioned methods.

To observe the prediction results of the different models, Fig. 6 shows the confusion matrices obtained by training on SVM, Xgboost, RF, GoogLeNet and PD-ResNet, respectively. SVM, RF, and Xgboost are excellent traditional ML algorithms that have been proved to perform well in classification tasks. The GoogLeNet algorithm is a state-of-the-art deep neural network model based on the inception module, inception is a sparse structure that can somewhat reduce overfitting. Fig. 6 also shows the confusion matrices of GoogLeNet and PD-ResNet with three different loss functions (namely the cross-entropy loss function, the focal loss function, and the improved focal loss function).

To compare the performance, Table 2 lists the accuracy, precision, recall, specificity, and F1-score of the different models according to their confusion matrices. It can be seen that PD-ResNet with improved focal loss function shows the best performance with an accuracy of 95.51%, a precision of 94.44%, a recall of 96.59%, a specificity of 94.44%, and an F1-score of 95.50%. PD-ResNet has a strong learning capability, and it solves the gradient problem very well. The accuracy of the proposed model can be improved by 3.38%-5.06% compared to three representative traditional ML algorithms. The reason is that the residual units are used to enhance the learning capability of the model. Furthermore, the accuracy of the proposed method can be improved by 3.38% compared to GoogLeNet with the cross-entropy loss function.

**TABLE 2:** Performance Comparisons of the Mentioned Methods

Method	Loss function	Accuracy	Precision	Recall	Specificity	F1-score
SVM	–	90.45%	90.91%	89.89%	91.01%	90.40%
Xgboost	–	92.13%	94.05%	89.77%	94.44%	91.86%
RF	–	91.01%	89.25%	93.26%	88.76%	91.21%
GoogLeNet	cross-entropy loss	92.13%	94.05%	89.77%	94.44%	91.86%
	focal loss	91.57%	91.01%	92.05%	91.11%	91.53%
	improved focal loss	91.57%	87.63%	96.59%	86.67%	91.89%
PD-ResNet	cross-entropy loss	93.26%	91.30%	95.45%	91.11%	93.33%
	focal loss	93.26%	94.19%	92.05%	94.44%	93.11%
	improved focal loss	95.51%	94.44%	96.59%	94.44%	95.50%

**FIGURE 7.**
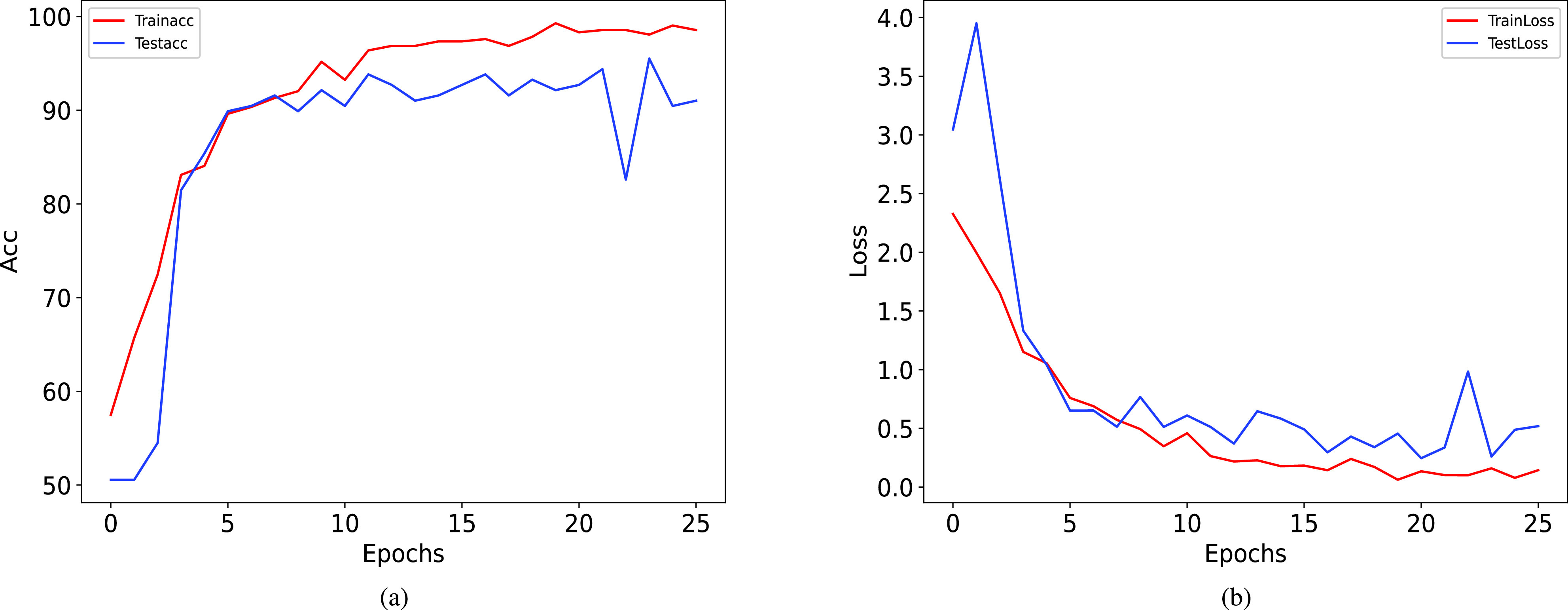
The dynamic change curves of accuracy and loss for the proposed method.

To observe the fitting degree of the proposed method, Fig. 7 plots the dynamic change curves of accuracy and loss on the training and test sets during the iteration process. From Fig. 7, it can be observed that as the number of iteration grows, the model’s accuracy exhibits an increasing trend while the loss value shows a decreasing trend. After 25 epochs, the proposed model reaches saturation, and training is stopped with the help of early stopping. It is easily seen that the fitting degree of the proposed method in the whole training and testing processes conforms to the basic principle, and there is no severe over-fitting risk.

**FIGURE 8.**
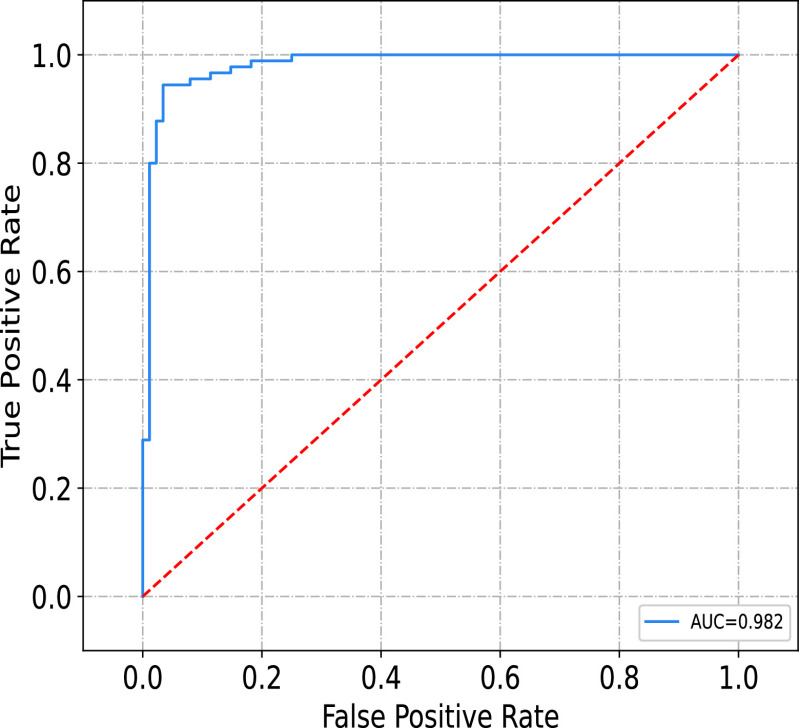
The ROC curve of the proposed method.

To observe the overall performance of the proposed method, the Receiver Operating Characteristic (ROC) Curve and Area Under Curve (AUC) of the predicted results of 178 test samples are shown in Fig. 8. The AUC reaches 0.982, which indicates the excellent performance of our model.

#### 2) Classification of Early PD and Moderate to Advanced PD

It is clinically significant to differentiate between early PD and moderate to advanced PD due to the different treatment schemes. Moreover, it can be seen from Table 1 that there is statistically significant differences between early PD and moderate to advanced PD for UPDRS. All 296 PD patients in our dataset were classified according to H-Y scores [23] into 230 patients with early PD and 66 patients with moderate to advanced PD. The 296 PD samples were individually removed as a new dataset, then the same dataset partition method was used and the PD-ResNet was adopted for training where all parameters were set in the same way. The accuracy, precision, recall, specificity, and F1-score are 92.03%, 94.29%, 90.41%, 93.85%, and 92.31%, respectively. These results indicate that the proposed method is also applicable to differentiate early PD from moderate to advanced PD.

#### 3) Classification of Early PD and HC

Since the symptoms of early PD are not obvious, it makes the diagnosis difficult. The 161 HCs and 230 early PD samples were moved out separately as a new dataset. The same data preprocess and dataset partition methods were used. Then PD-ResNet was adopted for training where all parameters were set in the same way. The accuracy, precision, recall, specificity, and F1-scores are 92.03%, 94.20%, 90.28%, 93.94%, and 92.20%, respectively, which indicates that the proposed method is also applicable to differentiate early PD from HC.

## IV. Discussion

Since traditional clinical assessment scales are subjective and expensive, it is of great importance to use advanced analytical techniques to identify PD in clinical practice. ML and DL methods are widely used for automatic quantification and motor impairment analysis in PD. In this paper, gait features are fed into the PD-ResNet based on the residual unit for training, and an improved focal loss function is proposed. PD-ResNet can efficiently extract and aggregate the richest gait features. Improved focal loss function inherits the advantages of focal loss function, which makes the network mainly focus on learning hard samples. Furthermore, the effect of anomalous samples is also taken into account by improved focal loss function. Our proposed PD-ResNet with improved focal loss function achieves good classification performance, and its accuracy, precision, recall, specificity, and F1-score are 95.51%, 94.44%, 96.59%, 94.44%, and 95.50%, respectively. The dynamic change curves of accuracy and loss from Fig. 7 show that the proposed method does not risk over-fitting. The proposed method is also suitable for the identification of early PD and moderate to advanced PD, and the accuracy, precision, recall, specificity, and F1-score are 92.03%, 94.29%, 90.41%, 93.85%, and 92.31%, respectively. Furthermore, the proposed method can also classify early PD and HC with an accuracy of 92.03%, a precision of 94.20%, a recall of 90.28%, a specificity of 93.94%, and an F1-score of 92.20%.

There are various ways to extract gait characteristics from PD patients. Since wearable sensor devices have the advantages of economy and objectivity, they are used for the automatic quantification of PD gait movement impairments. Table 3 in detail compares the differences between the proposed method and the existing methods for automatically quantifying the gait of PD patients using wearable sensor devices. Only the features on the lower back, two shank, and feet from the subjects were extracted in [7], [29], and [30], respectively. However, the gait of PD patients depends on various aspects of body coordination, such as the walking process on the lower limb requires coordination between the swing of the upper limb and the central trunk to maintain stability. The recognition results may be adversely affected due to few acquisition nodes, single action, and few features in the sensor acquisition system. Recently, the method on capturing multiple gait characteristics of the whole body by using numerous wearable sensor devices was proposed in [9]. The 134 gait features of the subjects under TUG test were extracted, and the PD was classified using traditional ML methods. In this paper, 194 comprehensive gait characteristics from subjects were extracted using the GYENNO MATRIX wearable device. To exclude the effect of coincidence, each subject was asked to test under three experimental environments, i.e., TUG, TURN, and NARROW. An end-to-end DL approach based on the resnet unit is used so that manual feature selection is not required. Moreover, an improved focal loss function is proposed, and some techniques such as data augmentation, polynomial elevated dimensions, early stopping, and LR decay are applied. Therefore, compared with these existing studies, the proposed method has the following advantages: 1) A considerable amount of data is available and a large number of features are extracted for each subject. 2) An end-to-end DL approach is adopted without requiring additional manual feature extraction.

**TABLE 3:** Comparison With the Existing Works

Paper/Year	Participants	Sensor device	Features	Experimental environment	Method
[29]/2013	20 early PD, 20 HC	a tri-axial accelerometer sensor	features in subjects’ lower back	iTUG	LDA, QDA and Mahalanobis classifier
[7]/2020	49 subjects	Inertial measurement units (IMU)	8 kinematic features about spatial-temporal in subjuects’ two shank	walk straight in preferred speed	SVM
[30]/2018	93 PD, 73 HC	foot sensors	features about spatial-temporal in subjuects’ feet	walk straight in their usual	a two-channel network including LSTM and CNN
[9]/2021	332 PD, 100 HC	cross-sectional wearable-sensor	134 comprehensive features including upper and lower body	TUG traditional	ML methods
ours	296 PD, 161HC	GYENNO MATRIX wearable device	194 comprehensive features including upper and lower body	TUG, TURN, NARROW	PD-ResNet

To obtain enough features for the training and type conversion of our dataset, in this paper, the polynomial elevated dimensions technology is adopted to enhance the dimensionality of the features of the input data. In this method, the interactive multiplication of feature data is utilized to increase the feature dimensionality. In this paper, the datasets before and after the polynomial boosting process were put into a logistic regression algorithm for training, respectively. The results show an accuracy of 86.95% for the former and 91.30% for the latter. Hence, it proves that the polynomial elevated dimensions technology can be fully applicable to the dataset of this paper.

Imbalanced samples may negatively affect the accuracy of the model and thus be detrimental to the training of the classification network. In this paper, sample balancing is achieved by using SMOTE. To demonstrate the effectiveness of the method, the differences in model performance before and after using SMOTE are presented in Table 4 It can be seen from the table that SMOTE improves the overall performance of the model.

**TABLE 4:** Performance of the Proposed Model With and Without SMOTE

Classification Task	SMOTE	Number of samples	Accuracy	Precision	Recall	Specificity	F1-score
PD and HC	No	296PD and 161HC	91.30%	88.64%	84.78%	94.57%	86.67%
	Yes	296PD and 296HC	95.51%	94.44%	96.59%	94.44%	95.50%
HC and early PD	No	161HC and 230 early PD	87.29%	88.89%	84.21%	90.16%	86.49%
	Yes	230HC and 230 early PD	92.03%	94.20%	90.28%	93.94%	92.20%
early PD and moderate to advanced PD	No	230 early PD and 66 moderate to advanced PD	86.52%	87.18%	97.14%	47.37%	91.89%
	Yes	230 early PD and 230 moderate to advanced PD	92.03%	94.29%	90.41%	93.85%	92.31%

From table 2 we can see that the improvement in performance comes mainly from the proposed method in the paper. Although our proposed method shows good performance, the present work has several limitations. Firstly, the evaluation was done ON medication, but the features under OFF-state maybe different for patients with advanced PD who suffer from severe motor impairment. Secondly, the collection of gait parameters from subjects is limited to the hospital ward and clinic environment which are very different from their real living conditions. Many psychological factors may affect the measurement results due to environmental disturbances. Hence, a system suitable for long-term monitoring in daily life conditions should be further developed. The system can collect the motor symptoms from PD patients anytime and anywhere without manufactured interference and transmit them remotely. It will provide researchers with remote monitoring data and realize remote home monitoring. Moreover, PD patients may also experience freezing of gait, i.e., FOG, which often occurs in their legs during walking [31]. However, in our study, due to the short duration of the test and the limited number of patients, a few patients who reported a FOG medical history did not present any FOG during testing. Finally, one of the main challenges in PD includes the differential diagnosis with other neurological disorders (e.g., progressive supranuclear palsy, essential tremor and multisystem atrophy) rather than HC only. Because the movement disorder-related symptoms of these diseases often show remarkable similarities, it is necessary to conduct an in-depth analysis of the movement signal to reduce the possibility of misjudgment.

Future work is recommended to further expand the dataset, which improves the classification accuracy and generalization ability. Hence, more accurate classification of the severity of PD patients can be realized with sufficient dataset.

## V. Conclusion

In this paper, a new PD-ResNet structure based on the resnet unit has been presented to realize the automatic recognition of PD and classify PD severity. With its excellent performance, PD-ResNet aggregates rich feature information and solves the gradient problem very well. Furthermore, an improved focal loss function has been proposed. The experiments show that the proposed PD-ResNet with improved focal loss function can efficiently identify PD. The proposed method has great potentials for the applications on intelligent diagnosis and medical automation in PD field, which can provide clinicians with effective help in diagnosing PD.

## Supplementary Materials

Supplementary materials
