# Supplementary material for: PD-ResNet for Classification of Parkinson’s Disease From Gait
Source: IEEE J Transl Eng Health Med. 2022 Jun 8;10:2200111. doi: 10.1109/JTEHM.2022.3180933 (PMC9252336; doi:10.1109/JTEHM.2022.3180933)
Supplement: Supplementary materials [file supp1-3180933.pdf]

GYENNO MATRIX[1] is a motor evaluation device that includes ten wireless MEMS inertial sensor nodes, a data center, and corresponding computer software. In this paper, subjects wore the wearable sensor device correctly with the help of a physician or their family members (see Fig. S1). They completed the standardized movements under the three test conditions of Time Up and GO (TUG)[2], TURN[2], and NARROW[3], respectively. The data from the sensors is transmitted in real-time to computer software via the data center, and the computer software completes the automatic quantification of human gait parameters. Each task was required to perform 2-3 times. Finally, the test results were averaged.

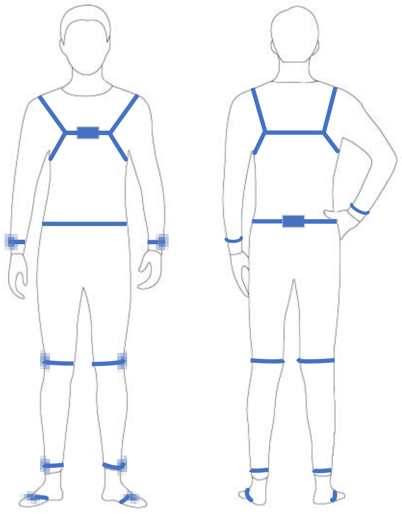

Fig. S1: Location of wearable sensor device

In this paper, each sample consists of 194 features: 4 demographic features (gender, age, length of thighs, and length of lower legs), 97, 7 and 86 features extracted in the TUG, TURN and NARROW tests, respectively. The relevant descriptions of these features are listed in Table S1. Similar features in different test cases appear only once in Table S1, e.g., 15 features for the turning process in the three test cases, 6 features for the time duration, and 156 features for the walking process in both the TUG and NARROW tests.

Table S1: Related description of sensor features

Category of the features	Features	Description of the features
demographic features (4)	Age	Age [years]
	Gender	Sex [male/female]
	Length of thighs	Length of thighs [cm]

	Length of lower legs	Length of lower legs [cm]
TUG test process Duration (6)	SiSt Duration	If it is multiple stand-up processes, the average time of these processes is calculated. [sec]
	Walking Duration (go)	If it is multiple forward walking processes, the average duration of these processes is calculated. [sec]
	Turning Duration （go）	If it is multiple turning processes, the average duration of these processes is calculated. [sec]
	Walking Duration (back)	If it is multiple return processes, the average duration of these processes is calculated. [sec]
	Turning Duration （back）	If it is multiple turning processes, the average duration of these processes is calculated. [sec]
	StSi Duration	If it is multiple sitting processes, the average duration of these processes is calculated. [sec]
Stand up process (4)	SiSt Duration	If it is multiple stand-up processes, the average duration of these processes is calculated. [sec]
	Trunk Sagittal Peak Velocity	If it is multiple stand-up processes, the average of the multiple trunk sagittal peak velocities is calculated. [degree/sec]
	Trunk Sagittal RoM Maximum	If it is multiple stand-up processes, the mean value of the maximum trunk back angle for these processes is calculated. [degree]
	Trunk Sagittal RoM Minimum	If it is multiple stand-up processes, the average value of the maximum forward trunk angle for these processes is calculated. [degree]

Walking process（78）	Step Length R.	Projection of the straight-line distance between the left-heel landing and the right-heel landing in the left gait cycle on the sagittal plane of the body. [cm]
	Step Length L.	Projection of the straight-line distance between the right-heel landing and the left-heel landing in the right gait cycle on the sagittal plane of the body. [cm]
	Step Length	Average of all left and right foot step lengths. [cm]
	Stride Velocity R.	Average of the ratio of all right stride step lengths to the cycle time of the current right stride. [m/s]
	Stride Velocity L.	Average of the ratio of all left stride step lengths to the cycle time of the current left stride. [m/s]
	Stride Velocity	The average velocity of all the left and right feet is calculated. [m/s]
	Stride Length R.	The distance (in a straight line) between two consecutive right-heel landings to the projection length of the sagittal plane of human motion. [cm]
	Stride Length L.	The distance (in a straight line) between two consecutive left-heel landings to the projection length of the sagittal plane of human motion. [cm]
	Stride Length	Average of left and right stride lengths. [cm]
	Gait Cycle R.	The time interval between two successive landings on the right heel. [sec]
	Gait Cycle L.	The time interval between two successive landings on the left heel. [sec]
	Gait Cycle	Average of all left and right stride durations. [sec]
	Cadence R.	Average of all right cadence in the straight line phase. [step/min]

	Cadence L.	Average of all left cadence in the straight line phase. [step/min]
	Cadence	Average of all mean left and right cadence in the straight-ahead phase. [step/min]
	Right Double Support	Percentage of the sum of the time from right-heel landing to left toe-off and from left-heel landing to right-toe off in a right gait cycle out of the total gait cycle time. [%]
	Left Double Support	Percentage of the sum of the time from left-heel landing to right toe-off and from right-heel landing to left-toe off in a left gait cycle out of the total gait cycle time. [%]
	Double Support	Average of all left and right leg supports. [%]
	Swing R.	Average of all right swing phases. [%]
	Swing L.	Average of all left swing phases. [%]
	Swing	Average of all left and right swing phases. [%]
	Stance R.	Average of all right support phases. [%]
	Stance L.	Average of all left support phases. [%]
	Stance	Average of all left and right support phases. [%]
	Shank RoM Maximum R.	Average of the maximum forward swing angle of all right legs when walking straight. [degree]
	Shank RoM Maximum L.	Average of the maximum forward swing angle of all left legs in a straight line. [degree]
	Shank RoM Maximum	Average of all left and right lower leg forward swing angle maxima in the straight line phase. [degree]

	Shank RoM Minimum R.	Average of the minimum backswing angle of the right lower leg in all straight phases. [degree]
	Shank RoM Minimum L.	Average of the minimum backswing angle of the left lower leg in all straight-ahead phases. [degree]
	Shank RoM Minimum	Average of the left and right lower leg backswing angle maxima for all straight-ahead phases. [degree]
	Peak Shank Angular Velocity R.	Average of the right lower leg angular velocity peaks during all straight-ahead phases. [degree/sec]
	Peak Shank Angular Velocity L.	Average of the left lower leg angular velocity peaks during all straight-ahead phases. [degree/sec]
	Peak Shank Angular Velocity	Average of the left and right lower leg angular velocity peaks for all straight-ahead phases. [degree/sec]
	Stride Velocity Asymmetry	The ratio of the absolute value of the difference between all left foot speeds and all right foot speeds to the maximum. [%]
	Stride Velocity Absolute Deviation	The absolute value of the difference between all left foot velocities and all right foot velocities. [m/s]
	Stride Length Asymmetry	The absolute value of the difference between the left and right stride lengths in all straight stages and the average of the larger percentage of the two. [%]
	Stride Length Absolute Deviation	The absolute value of the difference between the left and right stride lengths for all straight-ahead phases. [cm]
	Swing Asymmetry	Average of the relative deviations of all oscillating phases in both phases. [%]
	Swing Absolute Deviation	Average of the absolute deviations of the oscillation phase for all straight phases. [%]

	Stance Asymmetry	Average of the relative deviations of left and right leg supports in all straight phases. [%]
	Stance Absolute Deviation	Average of the absolute deviation of the left and right leg supports in all straight phases. [%]
	Shank RoM Asymmetry	Relative deviation in the angular range of the right and left lower leg is the ratio of the difference between the angular range of the two legs in absolute terms to the greater. [%]
	Shank RoM Absolute Deviation	The absolute deviation of the left and right lower leg angle range is the absolute value of the difference between the two leg angle ranges. [degree]
	Peak Shank Angular Velocity Asymmetry	The left lower leg peak angular velocity for each step of all straight phases minus the corresponding right lower leg peak angle, followed by the absolute value as a percentage of the greater values. [%]
	Peak Shank Angular Velocity Absolute Deviation	The absolute value of the average of the left lower legs' peak angular velocity per step minus the corresponding right lower leg's peak angle per step for all straight-ahead phases. [degree/sec]
	Shank Symbolic Symmetry Index	The average value of the symmetry index at each step for all straight-ahead phases. [%]
	Mean Phase Difference	The relative mean phase difference of the longer legs in the swinging phase. [%]
	Phase Coordination Index	The corresponding value of the gait cycle with a greater mean swing phase duration. [%]
	Trunk Coronal Peak Velocity	Average peak angular velocities in the coronal plane of the trunk in all straight-ahead phases. [degree/sec]

	Trunk Coronal RoM Maximum	The average of the maximum right tilt angle of the trunk during each gait cycle in all linear phases. [degree]
	Trunk Coronal RoM Minimum	The average of the maximum left tilt angle of the trunk during each gait cycle in all linear phases. [degree]
	Trunk Sagittal Peak Velocity	Average peak angular velocities of the trunk in the sagittal plane during all straight phases. [degree/sec]
	Trunk Sagittal RoM Maximum	Average of the maximum values of the trunk back angle in all straight phases. [degree]
	Trunk Sagittal RoM Minimum	Average of the maximum values of the forward trunk angle in all straight phases. [degree]
	Trunk Transverse Peak Velocity	Average peak angular velocities in trunk cross-sections during all straight phases. [degree/sec]
	Trunk Transverse RoM Maximum	Average of the maximum values of the right trunk angle in all straight phases. [degree]
	Trunk Transverse RoM Minimum	Average of the maximum values of the trunk left angle in all straight phases. [degree]
	Lumbar Coronal Peak Velocity	Average peak angular velocities in the coronal plane of the waist during all straight-ahead phases. [degree/sec]
	Lumbar Coronal RoM Maximum	Average of maximum waist-to-the-right angle per gait cycle for all straight phases.[degree]
	Lumbar Coronal RoM Minimum	Average of maximum waist-to-the-left angle per gait cycle for all straight phases. [degree]
	Lumbar Sagittal Peak Velocity	Average peak angular velocities of waist cross-sections for all straight-ahead phases. [degree/sec]

	Lumbar Sagittal RoM Maximum	Average of the maximum waist backward angle in all straight-ahead phases. [degree]
	Lumbar Sagittal RoM Minimum	Average of maximum waist forward angle in all straight phases. [degree]
	Lumbar Transverse Peak Velocity	Average of the peak angular velocities of the waist cross-section during all straight-ahead phases. [degree/sec]
	Lumbar Transverse RoM Maximum	Average of the maximum waist right rotation angle values in all straight phases. [degree]
	Lumbar Transverse RoM Minimum	Average of the maximum waist left rotation angle values in all straight phases. [degree]
	Arm Peak Velocity R.	Average right arm angular velocity maxima for each gait cycle in all straight-ahead phases. [degree/sec]
	Arm Peak Velocity L.	Average left-arm angular velocity maxima for each gait cycle in all straight-ahead phases. [degree/sec]
	Arm Peak Velocity	Average of the maximum angular velocities of the left and right arms for each gait cycle in all straight-ahead phases. [degree/sec]
	Arm RoM Maximum R.	Average right arm forward swing angle maxima for each gait cycle in all straight-ahead phases. [degree]
	Arm RoM Maximum L.	Average left-arm forward swing angle maxima for each gait cycle in all straight-ahead phases. [degree]
	Arm RoM Maximum	Average of the left and right arm forward swing angle maxima for each gait cycle in all straight-ahead phases. [degree]
	Arm RoM Minimum R.	Average of the maximum right arm backward swing angle per gait cycle in all straight phases. [degree]

	Arm RoM Minimum L.	Average of the maximum left-arm backward swing angle per gait cycle in all straight phases. [degree]
	Arm RoM Minimum	Average of the maximum left and right arm backward swing angles for each gait cycle in all straight-ahead phases. [degree]
	Arm Velocity Asymmetry	The percentage of the absolute value of the difference between the peak left-arm swing angle velocity and the peak right arm swing angle velocity and the greater for each gait cycle in all straight-ahead phases. [%]
	Arm Velocity Absolute Deviation	The absolute value of the difference between the left and right arm swing angular velocity peaks for each gait cycle in all straight phases. [degree/sec]
	Arm Symbolic Symmetry Index	Average arm angle range symmetry indices for each gait cycle in all straight-ahead phases. [%]
Turning process（7）	Turning Duration（L）	If there are multiple left-turn processes, the average time for each left-turn process is calculated. [sec]
	Turning Duration（R）	If there are multiple right-turn processes, the average time for each left-turn process is calculated. [sec]
	Average Duration	If there are multiple turning processes, the average time for each turning process is calculated. [sec]
	Average Steps	Average of step counts for multiple turning processes.
	Peak Velocity	Average of the peak angular velocities for multiple turning processes. [degree/sec]
	Average Angular Velocity	Average of turning angle divided by turning duration. [degree/sec]
	Average Step Duration	Average of the step durations for all turning processes. [sec]

Sitting process（4）	Average Duration	The average time of multiple sitting processes is calculated if it is multiple sitting processes. [sec]
	Trunk Sagittal Peak Velocity	If it is multiple sitting processes, the average of the multiple trunk peak angular velocities is calculated. [degree/sec]
	Trunk Sagittal RoM Maximum	If it is multiple sitting processes, the mean value of the trunk tilt angle maximum over the multiple sitting process is calculated. [degree]
	Trunk Sagittal RoM Minimum	If it is multiple sitting processes, the average torso forward angle maxima for multiple sitting processes is calculated. [degree]

Reference List

[1] Gyenno science, https://www.gyenno.com/matrix-en.

[2] L. Palmerini, S. Mellone, G. Avanzolini, F. Valzania, and L. Chiari, “Quantification of motor impairment in Parkinson’s disease using an instrumented timed up and go test,” *IEEE Trans. Neural Syst. Rehabil. Eng.*, vol. 21, no. 4, pp. 664–673, Jul. 2013.

[3] L. Rocchi, C. Minardi, M. Mancini, M. Dozza, F. Rasi, L. Chiari, “15.28 Kinematic assessment of walking through narrow spaces in subjects with Parkinson's disease,” *Gait & Posture*, vo1. 21, pp. S98, Jun. 2005.
